# Long-Term Impact of a Community Health Worker Intervention on Diabetes Control in American Samoa

**DOI:** 10.5888/pcd12.150160

**Published:** 2015-10-22

**Authors:** Mayuree Rao, Judith D. DePue, Shira Dunsiger, Mohammad Elsayed, Ofeira Nu'usolia, Stephen T. McGarvey

**Affiliations:** Author Affiliations: Mohammad Elsayed, Warren Alpert Medical School of Brown University, Providence, Rhode Island; Judith D. DePue, Shira Dunsiger, Warren Alpert Medical School of Brown University, Providence, Rhode Island, and Centers for Behavioral and Preventive Medicine, Miriam Hospital, Providence, Rhode Island; Ofeira Nu'usolia, Tafuna Clinic, American Samoa Community Health Centers, Department of Health, American Samoa; Stephen T. McGarvey, International Health Institute and Department of Epidemiology, Brown University School of Public Health, Providence, Rhode Island.

## Abstract

**Introduction:**

Diabetes Care in American Samoa (DCAS) was a randomized controlled trial of a 12-month intervention facilitated by community health workers (CHWs) that demonstrated improved HbA1c levels compared with usual care at trial completion. We sought to evaluate the long-term impact of this intervention on diabetes control.

**Methods:**

We retrospectively collected HbA1c measurements from medical records of DCAS participants (n = 268). The study group received the intervention during the trial, and the control group received the intervention after the trial. We used mixed-effects longitudinal regression models to assess change in HbA1c within each trial arm during 3 time periods: DCAS (12 months of the study group’s intervention), the first year after DCAS (control group’s intervention), and the second year after DCAS. Models were adjusted for baseline characteristics that differed significantly for participants with a low number of HbA1c measurements from those with a high number of HbA1c measurements.

**Results:**

After adjustment for confounders*,* the experiment group experienced a decrease in HbA1c of 0.28 units per year (95% confidence interval [CI], −0.64 to 0.07) during DCAS (intervention). HbA1c decreased by 0.88 units per year (95% CI, −1.31 to −0.45) during the year after the intervention. No significant change was observed the following year. HbA1c of the control group did not significantly change during DCAS (usual care) but decreased by 1.31 units per year (95% CI, −1.72 to −0.91) during its intervention. During the year after the control group’s intervention, HbA1c increased by 1.18 units per year (95% CI, 0.42 to 1.93).

**Conclusion:**

Both groups had initial improvements in glycemic control, but HbA1c later plateaued or increased. These results suggest that time-limited CHW programs improve diabetes control in the short term, but ongoing programs are needed for sustained impact.

## Introduction

Levels of type 2 diabetes in the US Territory of American Samoa are among the highest in the world, with an estimated prevalence of 21.5% in 2002 among adults aged 18 or older (1). The high prevalence of diabetes is associated with lifestyle changes accompanying economic modernization, including reduced physical activity and a shift to imported and highly processed foods (2,3). Furthermore, as a medically underserved area with a shortage of health professionals (4), American Samoa has limited health resources available for diabetes management.

Diabetes Care in American Samoa (DCAS) was a randomized controlled trial of a 12-month diabetes education and support program, culturally adapted for American Samoa and delivered by a team comprising a trained nurse and community health workers (CHWs). The experiment group was compared with a control group who received usual care and who also received the intervention after the study ended. At trial completion, HbA1c, a measure of average blood glucose, was 0.53 units lower in the experiment group than in the control group after we adjusted for confounders (5).

Most programs that use CHWs in diabetes care are time-limited (median duration: 6 months), and few follow up with participants long-term (6,7). The results of this study contribute to evidence supporting the effectiveness of CHWs in diabetes care (7–9). However, little is known about whether short-term improvements in diabetes control are sustained after CHW interventions are over (8).

**Figure 1:**
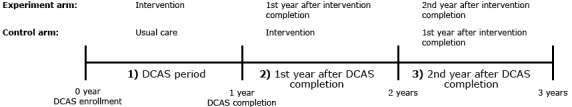
Timeline of study analysis. Change in HbA1c over time was evaluated by trial arm during 3 consecutive 1-year periods: 1) DCAS period; 2) first year after DCAS completion; and 3) second year after DCAS completion. Abbreviation: DCAS, Diabetes Care in American Samoa.

Here, we report the longer-term impact of our 12-month CHW intervention on diabetes control. Our objective was to evaluate the change in HbA1c by trial arm for 3 consecutive 1-year periods (Figure 1). We hypothesized that HbA1c would increase over time in the experiment group after the intervention ended. For the control group, we expected HbA1c to decrease during the intervention and increase after its completion.

## Methods

### Study design, setting, and participants

DCAS was a cluster randomized controlled trial conducted in Tutuila, the main island in American Samoa. Details are discussed elsewhere (5). Briefly, the study design and intervention was adapted from Project Sugar 2, a nurse-CHW team intervention for diabetes management for African Americans in Baltimore, Maryland (9,10). DCAS participants were drawn from the patient population of Tafuna Clinic, a federally qualified community health center. Villages within the clinic’s catchment area were divided into 12 areas. To limit contamination, villages were randomized to the experiment or control group on the basis of size and location so that intervention and usual-care villages were not adjacent. As part of the study design, the control group also received the CHW intervention following the completion of the trial.

DCAS participants were enrolled by Tafuna Clinic staff on a rolling basis from February 2009 to May 2010. Eligibility criteria were broad to test real-world effectiveness: aged 18 or older, resident in service area, self-identified as Samoan, type 2 diabetes diagnosed by a physician, mentally competent and able to give informed consent, unlikely to leave American Samoa for more than 4 months during the study, and having no serious comorbid conditions (eg, end-stage renal disease, cancer). Because recruitment initially yielded fewer patients with diagnosed diabetes than expected, Tafuna Clinic providers extended the criteria to include newly diagnosed patients after community diabetes screenings and confirmation of diabetes diagnosis.

**Figure 2:**
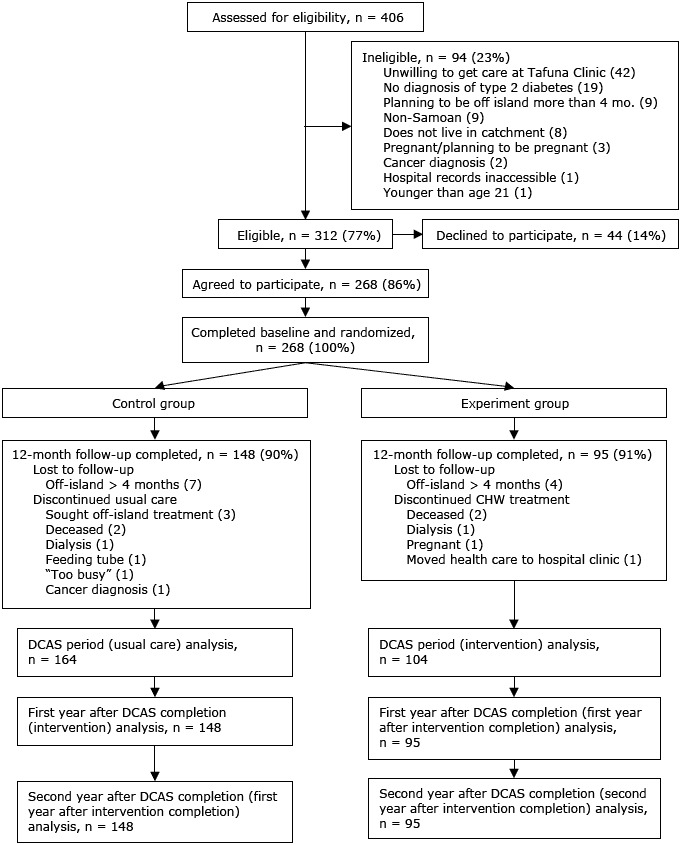
Consolidated Standards of Reporting Trial (CONSORT) diagram of recruitment. In this intent-to-treat analysis, participants who did not complete 12-month follow-up were included in analysis until date of ineligibility. Abbreviations: CHW, community health worker; DCAS, Diabetes Care in American Samoa.

Our study sample comprised all 268 participants who were enrolled in DCAS: 104 assigned to the experiment group and 164 to the control group (Figure 2). The primary outcome measure was HbA1c, collected retrospectively from the medical records of DCAS participants. After the parent study (DCAS trial) ended, medical records were used because additional study measurements could not be collected in person.

Because the control group received the intervention after the experiment group did, it was not possible to use a randomized design for this follow-up study. Therefore, each group was compared with itself over time. All study protocols and informed consent procedures were approved by the institutional review boards of the American Samoa Department of Health and Brown University.

### DCAS intervention

The DCAS staff consisted of a nurse care manager and 4 CHWs. CHWs were recruited from the community through the Tafuna Clinic employee hiring system; the position required a high school education with some health care experience preferred. All CHWs were trained by study investigators, including a primary care physician, registered nurse and diabetes educator, and behavioral interventionist. Training included research practice, standards of care including American Diabetes Association guidelines (11), the chronic care model (12), and patient-centered communication skills (13). CHWs were further certified in diabetes knowledge and in procedures for taking blood glucose, blood pressure, and height and weight measurements.

Study participants were classified into 1 of 3 risk profiles, which determined intervention frequency and intensity. Risk profiles were based on an algorithm that used HbA1c, blood pressure, smoking status, alcohol use, and Patient Health Questionnaire (PHQ-9) depression scores measured at study enrollment. BMI was not included in the risk profile, given that 93.5% of American Samoans are overweight or obese (14). Low-risk participants received a one-on-one home CHW visit every 3 months, moderate-risk participants received a one-on-one CHW home visit monthly, and high-risk participants had weekly group meetings with the nurse care manager and CHW, or one-on-one meetings with a CHW if they were unavailable for group meetings.

Intervention content included 8 possible topics chosen from the American Association of Diabetes Educators’ selected self-care behaviors: diabetes introduction, healthy eating, physical activity, medication adherence, monitoring blood glucose and blood pressure, reducing risk, healthy coping, and problem-solving (15). Topic selection was guided by the risk profile and self-selected goals of the participants. CHWs used flipcharts including sections on each of the 8 topics to facilitate diabetes education during visits with participants. Flipcharts were modeled on the National Diabetes Education Program flipcharts for diabetes prevention and adapted for the local cultural context (16,17). Given the intervention time frame and limited scope of the CHWs’ training, weight loss was not directly targeted but indirectly addressed by topics such as healthy eating and physical activity.

### Data collection

At DCAS enrollment, information was collected on sociodemographic variables (eg, age, sex), biological measures (eg, HbA1c, BMI, blood pressure), behavioral measures (eg, diet, exercise), and psychosocial measures (eg, personal beliefs on diabetes control). After this baseline assessment, the experiment group received the 12-month CHW intervention while the control group received usual care, including any regularly scheduled primary care appointments. The biological, behavioral, and psychosocial measures of both groups were assessed 12 months after baseline assessment. After the follow-up assessment, the control group began receiving the CHW intervention. The data collected at baseline and 12 months after baseline were not collected again at the end of the control group’s intervention.

For this study on diabetes control over time, HbA1c measurements were collected retrospectively from the medical records of DCAS participants from the period between DCAS enrollment and June through August 2013. Because of rolling enrollment, the period of data collection after the experiment arm and the control arm completed the intervention ranged from 12.5 to 28.0 months for the control group and 32.9 to 41.2 months for the experiment group. HbA1c measurements were abstracted from 2 sources: 1) paper medical records at Tafuna Clinic and 2) the Computerized Patient Record System at Lyndon B. Johnson (LBJ) Tropical Medical Center, the only hospital in American Samoa, which provides emergency, acute, and specialty care. Although participants may have also obtained care at one of the other community health centers on the island or outside American Samoa, it is unlikely, given the great distances involved in getting such care.

### Statistical analysis

To determine whether there were any confounding factors related to frequency of HbA1c measurement, we compared baseline demographic, biological, behavioral, and psychosocial variables of participants who had a low number of HbA1c measurements with those of participants who had a high number of measurements within each trial arm, including the baseline and 12-month follow-up HbA1c levels measured as part of the DCAS trial. Differences were assessed between participants with less than 3 versus 3 or more HbA1c measurements during DCAS; almost all participants had at least 2 measurements taken as part of the trial. During subsequent periods, differences were assessed between participants with less than 2 versus 2 or more HbA1c measurements. Because demographic variables, risk classification, and comorbid conditions were measured only once (at DCAS enrollment), these enrollment measures were used in the analyses of data from all periods. For all other variables, DCAS enrollment measurements were used in the DCAS period analysis, and the 12-month follow-up measurements were used in the analyses of subsequent periods. Nonparametric and parametric tests were used as appropriate (eg, *t* test or Wilcoxon rank-sum test for continuous variables and χ^2^ or Fisher’s exact test for categorical variables). The mean number of HbA1c measurements by period was also calculated for each trial arm.

We used mixed-effects longitudinal regression models to assess whether HbA1c levels changed over time, separately for each trial arm, during each of the three 1-year periods described previously. Models were controlled for all baseline variables that were found to be significantly different between participants with a low number of HbA1c measurements and those with a high number in any period. Age was included as a covariate in all models because of its relationship with diabetes control. All models included random intercepts to account for within-subject correlation between repeated outcomes over time, and standard errors were adjusted to account for the 2-level clustering of households within villages. All models used an intent-to-treat analysis, including data from all participants randomized at DCAS enrollment until the date of loss to follow-up or discontinuation of care. All analyses were conducted in Stata version 12.1 (StataCorp, College Station, Texas) using command *xtmixed* with two-tailed α = 0.05.

## Results

Of eligible participants, 86% were enrolled in DCAS, and the trial had a 91% retention rate, with no difference in loss-to-follow-up between the experiment and control groups. Nine participants in the experiment group were lost to follow-up, and 16 in the control group during DCAS (Figure 2). For our analysis, the follow-up period was 3 years from DCAS enrollment (2 years postintervention completion for the experiment group and 1 year postintervention completion for the control group).

### Baseline characteristics

For both trial arms, mean age was 55 (SD, 12.7) years, 62% were female, 41% were unemployed, and mean years of education was 12.5 (SD, 2.2). Sociodemographic variables were similar for the experiment and control groups, with the exception of church attendance location; a greater percentage of participants in the experiment group attended church in a village randomized to the opposite trial arm. Church attendance was recorded to measure potential contamination between trial arms because participants in opposite trial arms who attended the same church may have discussed their participation in the study with each other. Clinical characteristics (HbA1c, risk group, BMI, blood pressure, waist circumference, and comorbid conditions) were similar for both groups. The mean baseline HbA1c level for both trial arms was 9.8 (SD, 2.2). Baseline characteristics by trial arm are fully reported elsewhere (5).

In the experiment group, participants with 3 or more HbA1c measurements during the DCAS (intervention) period had higher mean HbA1c level at DCAS enrollment and were more likely to be in the high-risk group than participants with fewer than 3 HbA1c measurements (Table 1). During the first year after intervention completion, participants with 2 or more HbA1c measurements were less likely to attend church in a village randomized to the control group, had higher mean diastolic blood pressure at DCAS enrollment, and had a higher number of primary care visits during the DCAS period. During the second year after intervention completion, participants with 2 or more HbA1c measurements had higher mean BMI and a higher number of primary care visits.

**Table 1:** Baseline Characteristics of Experiment Group by Number of HbA1c Measurements, Diabetes Control Intervention, American Samoa, 2009–2013

Variables	DCAS Study Period (Intervention)^a^			First Year After Intervention Completion^b^			Second Year After Intervention Completion		
Number of HbA1c Measurements	< 3	≥ 3	Total	< 2	≥ 2	Total	< 2	≥ 2	Total
Mean no. of HbA1c measurements (95% CI)	2.8 (2.6–3.0)			1.7 (1.5–1.9)			0.9 (0.7–1.1)		
No. of participants	46	58	104	47	48	95	69	26	95
**Demographics^c^**									
Age, mean (SD)	54.9 (12.6)	56.4 (12.4)	55.8 (12.5)	55.4 (12.4)	56.1 (12.0)	55.8 (12.2)	55.8 (11.9)	55.7 (13.1)	55.8 (12.2)
Married/with partner, %	82.6	75.9	78.9	78.7	79.2	79.0	76.8	84.6	79.0
Education years, mean (SD)	12.5 (2.1)	12.6 (2.4)	12.6 (2.3)	12.8 (2.5)	12.4 (2.1)	12.6 (2.3)	12.5 (2.3)	12.8 (2.5)	12.6 (2.3)
Females, %	60.9	53.5	56.7	55.3	60.4	57.9	63.8	42.3	57.9
Employed, %	46.7	41.4	43.7	55.3	37.5	46.3	44.9	50.0	46.3
Church attendance in opposite trial-arm village, %^d^	84.8	75.9	79.8	89.4^e^	68.8^e^	79.0	78.3	80.8	79.0
**Biological measures**									
HbA1c (%), mean (SD) **f**	8.9 (1.7)^e^	10.1 (2.2)^e^	9.6 (2.1)	9.4 (2.0)	9.1 (1.9)	9.3 (1.9)	9.1 (1.9)	9.6 (2.2)	9.3 (1.9)
Current daily smoker, %^f^	10.9	8.6	10.9	14.9	6.3	10.5	10.1	11.5	10.5
Depression symptoms/Patient health questionnaire-9, mean (SD)^f^	2.4 (2.0)	2.7 (2.7)	2.4 (2.0)	1.6 (0.85)	1.7 (1.0)	1.6 (0.96)	1.6 (0.97)	1.7 (0.93)	1.6 (0.96)
Algorithm risk level: low (quarterly visits by CHW)^c^	10.9	8.6	10.9	10.6	6.3	8.4	7.3	11.5	8.4
Algorithm risk level: moderate (monthly visits by CHW)^c^	63.0^e^	37.9^e^	63.0	42.6	58.3	50.5	55.1	38.5	50.5
Algorithm risk level: high (weekly visits by CHW)^c^	26.1^e^	53.5^e^	26.1	46.8	35.4	41.1	37.7	50.0	41.1
Body mass index, kg/m^2^, mean (SD)^f^	35.6 (6.1)	35.7 (6.9)	35.6 (6.1)	35.6 (5.8)	36.5 (7.0)	36.0 (6.3)	35.1 (5.9)^e^	38.6 (7.0)^e^	36.0 (6.3)
Systolic blood pressure, mean (SD)^f^	133 (15.5)	131 (18.8)	133 (15.5)	130 (16.4)	133 (12.4)	132 (14.5)	131 (14.4)	134 (14.6)	132 (14.5)
≥ 140 (%)^f^	32.6	36.2	32.6	25.5	35.4	30.5	27.5	38.5	30.5
Diastolic blood pressure, mean (SD)^f^	85 (6.6)	83 (8.6)	85 (6.6)	79 (9.5)^e^	84 (8.1)^e^	82 (9.0)	81 (9.0)	82 (9.2)	82 (9.0)
≥ 90 (%)^f^	23.9	24.1	23.9	17.0	29.2	23.2	21.7	26.9	23.2
Waist circumference, mean (SD)^f^	119.0 (19.9)	117.3 (18.1)	119.0 (19.9)	118.7 (9.4)	120.1 (13.9)	119.4 (11.8)	118.6 (11.4)	121.5 (12.9)	119.4 (11.8)
Any comorbid condition (% any)^c^	17.4	13.8	17.4	8.5	20.8	14.7	13.0	19.2	14.7
Medication adherence, %	46.7	35.7	46.7	76.6	85.4	81.1	84.1	73.1	81.1
Physical activity level (moderate/high, ≥ 600 metabolic equivalent minutes/week), %	39.1	50.0	39.1	70.2	64.6	67.4	69.6	61.5	67.4
Primary care visits, mean (SD)^g^	4.8 (3.5)	4.3 (3.9)	4.5 (3.7)	3.9 (2.7)^e^	7.5 (6.6)^e^	5.7 (5.4)	4.9 (4.2)^e^	7.7 (7.4)^e^	5.7 (5.4)
**Selected psychosocial variables^f^**									
Benefits of diabetes control (0**–**5 scale), mean (SD)	4.6 (0.48)	4.5 (0.50)	4.6 (0.48)	4.3 (0.40)	4.3 (0.36)	4.3 (0.38)	4.3 (0.37)	4.4 (0.40)	4.3 (0.38)
Patient Activation Measure (0**–**100 score), mean (SD)	83.8 (18.9)	83.0 (19.0)	83.8 (18.9)	78.2 (16.0)	73.8 (15.4)	76.0 (15.7)	76.2 (16.1)	75.5 (15.1)	76.0 (15.7)

Abbreviations: CHW, community health worker; CI, confidence interval; DCAS, Diabetes Care in American Samoa; HbA1c, a measure reflecting average blood glucose; SD, standard deviation.

a Includes baseline and 12-month follow-up HbA1c levels measured as part of DCAS study.

b Includes 12-month follow-up HbA1c levels measured as part of DCAS study.

c Measured at DCAS enrollment.

d Indicator of potential for contamination across trial arms by church attendance.

e Significant difference by HbA1c measurement category, *P* < .05. Nonparametric and parametric tests were used as appropriate (eg, *t* test or Wilcoxon rank-sum test for continuous variables and χ^2^ or Fisher’s exact test for categorical variables).

f DCAS enrollment measurement used in DCAS study period analyses. DCAS 12-month follow-up measurement used in subsequent time periods.

g Number of primary care visits during one year before DCAS enrollment used in DCAS study period analysis. Number of primary care visits during DCAS study period used in subsequent time periods.

Fewer differences were found between participants in the control group with a high number of HbA1c measurements and those with a low number. No differences were statistically significant during the DCAS (usual care) period, but participants with 2 or more HbA1c measurements during their intervention period had smaller abdominal circumferences at DCAS follow-up than did participants with fewer than 2 HbA1c measurements (Table 2). During the first year after their intervention completion, participants with 2 or more HbA1c measurements were more likely than those with fewer than 2 to have a comorbid condition.

**Table 2:** Baseline Characteristics of Control Group by Number of HbA1c Measurements, Diabetes Control Intervention, American Samoa, 2009–2013

Characteristics at Baseline	DCAS Study Period (Usual Care)^a^			Intervention Period^b^			First Year After Intervention Completion		
Number of HbA1c Measurements	< 3	≥ 3	Total	< 2	≥ 2	Total	< 2	≥ 2	Total
Mean number of HbA1c measurements (95% CI)	2.6 (2.5–2.7)			1.8 (1.6–2.0)			0.7 (0.6–0.8)		
Number of participants	90	74	164	66	82	148	116	32	148
**Demographics^c^**									
Age, mean (SD)	54.2 (12.2)	54.5 (13.7)	54.2 (12.2)	55.3 (13.5)	54.0 (12.7)	55.3 (13.5)	54.8 (14.0)	53.6 (8.8)	54.6 (13.0)
Married/with partner, %	77.8	78.4	77.8	77.3	78.1	77.3	75.0	87.5	77.7
Education years, mean (SD)	12.3 (2.6)	12.6 (1.7)	12.3 (2.6)	12.3 (2.2)	12.7 (1.7)	12.3 (2.2)	12.4 (1.9)	12.9 (2.1)	12.5 (1.9)
Females, %	66.7	63.5	66.7	65.2	65.9	65.2	69.0	53.1	65.5
Employed, %	36.7	44.6	36.7	40.9	42.7	40.9	37.9	56.3	41.9
Church attendance in opposite trial-arm village, %^d^	43.3	51.4	43.3	36.4	52.4	36.4	48.3	34.4	45.3
**Biological measures**									
HbA1c (%), mean (SD)^e^	9.8 (2.2)	10.1 (2.3)	10.0 (2.3)	9.7 (2.2)	10.2 (2.3)	10.0 (2.3)	9.9 (2.3)	10.4 (2.1)	10.0 (2.3)
Current daily smoker, %^e^	6.7	6.8	6.7	10.6	12.2	11.5	12.1	9.4	11.5
Depression symptoms/Patient Health Questionnaire-9, mean (SD)^e^	2.4 (1.9)	2.1 (1.3)	2.4 (1.9)	2.2 (2.4)	1.8 (1.4)	2.0 (1.9)	1.9 (1.8)	2.2 (2.2)	2.0 (1.9)
Algorithm risk level: low (quarterly visits by CHW)^c^	13.3	10.8	13.3	12.1	12.2	12.2	11.2	15.6	12.2
Algorithm risk level: moderate (monthly visits by CHW)^c^	37.8	36.5	37.8	37.9	35.4	36.5	33.6	46.9	36.5
Algorithm risk level: high (weekly visits by CHW)^c^	48.9	52.7	48.9	50.0	52.4	51.4	55.2	37.5	51.4
Body mass index, kg/m^2^, mean (SD)^e^	36.7 (7.7)	35.7 (7.9)	36.7 (7.7)	37.9 (7.6)	35.3 (7.9)	36.4 (7.9)	36.6 (7.9)	36.0 (7.9)	36.4 (7.9)
Systolic blood pressure, mean (SD)^e^	136 (18.8)	131 (15.4)	136 (18.8)	136 (15.8)	133 (14.1)	135 (14.9)	134 (15.1)	136 (14.3)	135 (14.9)
≥ 140, %^e^	43.3	37.8	43.3	43.9	29.3	35.8	32.8	46.9	35.8
Diastolic blood pressure, mean (SD)^e^	85.4 (11.8)	82.1 (9.9)	85.4 (11.8)	84 (10.0)	83 (10.2)	83 (10.1)	83 (10.3)	85 (9.3)	83 (10.1)
≥ 90, %^e^	40.0	28.4	40.0	30.3	29.3	29.7	26.7	40.6	29.7
Waist circumference, mean (SD)^e^	121.7 (15.4)	120.3 (17.9)	121.7 (15.4)	125.6 (19.7)^f^	117.6 (12.9)^f^	121.2 (16.7)	121.1 (15.9)	121.6 (19.7)	121.2 (16.7)
Any comorbid condition, %^c^	12.2	8.1	12.2	7.6	9.8	8.8	5.2^f^	21.9^f^	8.8
**Behavioral measures^e^**									
Medication adherence, %	54.4	59.5	54.4	56.9	56.3	56.6	56.6	56.3	56.6
Physical activity level (moderate/high, ≥ 600 metabolic equivalent minutes/week), %	46.7	52.7	46.7	51.5	37.8	43.9	44.8	40.6	43.9
Primary care visits, mean (SD)^g^	4.3 (3.3)	4.2 (3.9)	4.3 (3.3)	4.2 (3.5)	4.6 (4.1)	4.4 (3.9)	4.1 (3.6)	5.4 (4.6)	4.4 (3.9)
**Selected psychosocial variables^e^**									
Benefits of diabetes control (0–5 scale), mean (SD)	4.2 (0.44)	4.3 (0.46)	4.2 (0.44)	4.2 (0.28)	4.1 (0.32)	4.2 (0.30)	4.2 (0.32)	4.1 (0.26)	4.2 (0.30)
Patient Activation Measure (0–100 score), mean (SD)	75.2 (19.5)	73.7 (18.6)	75.2 (19.5)	70.2 (14.0)	68.2 (14.0)	69.1 (14.0)	69.2 (14.2)	68.7 (13.3)	69.1 (14.0)

Abbreviations: CHW, community health worker; CI, confidence interval; DCAS, Diabetes Care in American Samoa; HbA1c, a measure of average blood glucose; SD, standard deviation.

a Includes baseline and 12-month follow-up HbA1c levels measured as part of DCAS study.

b Includes 12-month follow-up HbA1c levels measured as part of DCAS study.

c Measured at DCAS enrollment.

d Indicator of potential for contamination across trial arms by church attendance.

e DCAS enrollment measurement used in DCAS study period analyses; DCAS 12-month follow-up measurement used in subsequent time periods.

f Significant difference by HbA1c measurement category, *P* < .05. Nonparametric and parametric tests were used as appropriate (eg, *t* test or Wilcoxon rank-sum test for continuous variables and χ^2^ or Fisher’s exact test for categorical variables).

g Number of primary care visits during one year before DCAS enrollment used in DCAS study period analysis; number of primary care visits during DCAS study period used in subsequent time periods.

### HbA1c level over time

For the experiment group, HbA1c decreased during DCAS (intervention), but the effect was not statistically significant (−0.28 units per year; 95% CI, −0.64 to 0.07) (Table 3). During the first year after intervention completion, HbA1c of the experiment group decreased further by 0.88 units per year (95% CI, −1.31 to −0.45). No significant change was observed during the second year after intervention completion (0.18 units per year; 95% CI, −0.77 to 1.13).

**Table 3:** Regression Coefficients From Mixed-Effects Regression Models for HbA1c Over Time, Diabetes Control Intervention, American Samoa, 2009–2013

Variable	Model, Experiment Group			Model, Control Group		
	DCAS Study Period (Intervention)^a^	First Year After Intervention Completion^b^	Second Year After Intervention Completion	DCAS Study Period (Usual Care)^a^	Intervention Period^b^	First Year After Intervention Completion
No. of participants in model n (HbA1c measurements)	96 (271)	90 (162)	52 (91)	159 (414)	143 (286)	71 (113)
Intercept, β (95% CI)	3.67 (0.09 to 7.23)	4.95 (2.02 to 7.88)	12.47 (4.03 to 20.91)	2.11 (0.24 to 3.98)	0.11 (−1.89 to 2.10)	8.47 (4.76 to 12.18)
Time (years), β (95% CI)	−0.28 (−0.64 to 0.07)	−0.88 (−1.31 to −0.45)	0.18 (−0.77 to 1.13)	0.01 (−0.24 to 0.26)	−1.31 (−1.72 to −0.91)	1.18 (0.42 to 1.93)
Age, β (95% CI)^c^	−0.02 (−0.04 to −0.01)	−0.01 (−0.02 to 0.01)	−0.07 (−0.10 to −0.04)	−0.01 (−0.02 to 0.004)	−0.004 (−0.02 to 0.01)	−0.02 (−0.05 to 0.004)
Church attendance in opposite village, β (95% CI)^c^	0.12 (−0.37 to 0.61)	−0.23 (−0.58 to 0.12)	−0.62 (−1.64 to 0.40)	0.10 (−0.17 to 0.37)	0.07 (−0.24 to 0.38)	0.53 (−0.04 to 1.09)
Baseline HbA1c, β (95% CI)^d^	0.64 (0.48 to 0.80)	0.72 (0.61 to 0.82)	0.66 (0.38 to 0.94)	0.77 (0.67 to 0.87)	0.80 (0.71 to 0.90)	0.38 (0.22 to 0.55)
Low risk, β (95% CI)^c^	−0.04 (−1.01 to 0.92)	−0.27 (−0.87 to 0.32)	−0.27 (−1.66 to 1.11)	−0.17 (−0.85 to 0.51)	−0.05 (−0.63 to 0.52)	−0.99 (−2.11 to 0.13)
Moderate risk, β (95% CI)^c^	0.13 (−0.48 to 0.74)	−0.16 (−0.49 to 0.18)	0.05 (−0.83 to 0.94)	−0.06 (−0.53 to 0.40)	−0.01 (−0.44 to 0.42)	−0.94 (−1.70 to −0.18)
Primary care visits, β (95% CI)^e^	−0.02 (−0.08 to 0.03)	−0.01 (−0.04 to 0.01)	−0.01 (−0.07 to 0.05)	−0.01 (−0.05 to 0.03)	0.01 (−0.04 to 0.05)	−0.10 (−0.19 to −0.02)
BMI, β (95% CI)^d^	−0.001 (−0.04 to 0.04)	0.003 (−0.03 to 0.03)	−0.08 (−0.17 to 0.01)	−0.02 (−0.04 to 0.001)	0.001 (−0.02 to 0.03)	0.07 (0.03 to 0.11)
Diastolic blood pressure, β (95% CI)^d^	0.01 (−0.01 to 0.04)	−0.003 (−0.02 to 0.01)	−0.02 (−0.06 to 0.02)	0.01 (−0.01 to 0.02)	−0.004 (−0.02 to 0.01)	−0.03 (−0.06 to 0.0004)
Waist circumference, β (95% CI)^d^	−0.004 (−0.02 to 0.01)	−0.01 (−0.03 to 0.01)	−0.01 (−0.06 to 0.05	0.01 (−0.01 to 0.02)	0.01 (−0.01 to 0.02)	−0.02 (−0.04 to 0.01)
Any comorbid condition, β (95% CI)^c^	0.41 (−0.13 to 0.95)	−0.17 (−0.57 to 0.22)	−0.25 (−1.51 to 1.00)	0.21 (−0.28 to 0.70)	0.46 (−0.05 to 0.96)	0.58 (−0.21 to 1.36)

Abbreviations: β, regression coefficient; CI, confidence interval; DCAS, Diabetes Care in American Samoa; HbA1c, a measure of average blood glucose.

a Includes baseline and 12-month follow-up HbA1c levels measured as part of DCAS study.

b Includes 12-month follow-up DCAS study HbA1c measurements.

c Measured at DCAS enrollment.

d DCAS enrollment measurement included in DCAS study period models. DCAS 12-month follow-up measurement included in subsequent models.

e Number of primary care visits during one year before DCAS enrollment used in DCAS study period model. Number of primary care visits during DCAS study period used in subsequent models.

For the control group, HbA1c did not significantly change over time during DCAS (usual care) but decreased by 1.31 units per year (95% CI, −1.72 to −0.91) during the intervention. During the first year after the control group’s intervention completion, HbA1c increased by 1.18 units per year (95% CI, 0.42 to 1.93).

## Discussion

We found that the experiment group experienced a nonsignificant decrease of 0.28 units per year during its intervention period. Notably, our previously published analysis found that the experiment group’s mean HbA1c after adjustment for confounding factors was 0.53 units lower than the control group’s HbA1c at 12-month follow-up. However, this study does not compare the 2 trial arms but rather examines the effect of time on each trial arm separately. Thus, the adjusted point estimate of −0.28 units per year is consistent with our prior analysis, finding an unadjusted decline in mean HbA1c of 0.3 units from baseline to 12-month follow-up for the experiment group (5). Furthermore, the upper confidence limit may be explained by the reduction in statistical power caused by analyzing each trial arm separately. The significant decline in HbA1c by 0.88 units during the first year after intervention completion suggests a continued and stronger effect of the CHW program even with no continued CHW contact. During the second year after intervention completion, HbA1c level stabilized, neither increasing nor decreasing further.

HbA1c of the control group decreased by 1.31 units during its intervention year, a stronger effect than that observed for the experiment group. Yet, unlike the experiment group, HbA1c levels of the control group all but rebounded during the year after its intervention completion; the control group did not experience the sustained, but temporary, intervention effect observed in the experiment group. Numerous factors may explain the different effects on each trial arm. Having already delivered the intervention to the experiment group, the CHWs were more experienced and therefore perhaps more effective during the control group’s intervention. Additionally, the control group may have been anticipating their turn with the intervention, thus increasing their motivation for behavioral change. On the other hand, CHWs were no longer closely supervised by DCAS investigators after the 12-month follow-up assessment, potentially impairing the long-term impact of the intervention on the control group. Furthermore, after the control group’s intervention, the nurse-CHW team was disbanded because research funding ended, perhaps contributing to the increase in HbA1c in the year of observation after the control group’s intervention.

The strengths of our study include the follow-up time of 3 years after DCAS enrollment, given that few studies of culturally appropriate diabetes education programs follow participants long-term (6). Furthermore, the enrollment of 86% of those eligible and 91% study retention support the generalizability of this study to the population of diabetes patients in American Samoa; we also believe these results may be relevant to other resource-poor populations with high rates of diabetes.

This study had some limitations. First, participants had different numbers of HbA1c measurements at different frequencies, compromising the reliability of this study. Second, given that the control group received the intervention after the experiment group did, it was not possible to assess the long-term impact of the intervention using a randomized design. Third, the level of social support available to participants was not measured and may have influenced the outcomes of our study. Fourth, participants were not assessed for changes in eligibility (eg, initiation of dialysis, travel off-island for more than 4 months) after the completion of the DCAS trial. Fifth, it is possible that some HbA1c measurements were not documented in the records from LBJ Hospital and Tafuna Clinic, but there was no way of determining the completeness or accuracy of the records. Sixth, HbA1c measurements taken at other facilities or off-island were not collected, but it is unlikely that many participants received care at other locations given the distance and cost involved in going to another location. Finally, this study may not be generalizable to non-Samoan populations, but we believe the results have relevance for other settings with a high prevalence of diabetes and low resources. Directions for future research include analyzing the long-term impact of the CHW intervention on health care use and other biologic factors such as BMI and blood pressure.

This study examined the long-term impact of a 12-month nurse-CHW intervention on diabetes control over time in American Samoa, a resource-poor setting with a high-risk population. Each trial arm experienced initial improvements in glycemic control during its intervention period, followed by either an increase or plateau of HbA1c levels. This study contributes to the literature that evaluates the effectiveness of CHWs in improving diabetes outcomes in resource-poor settings and explores the long-term impact of a limited-duration program. Our results suggest that continued improvements in diabetes control may require standing CHW programs fully integrated with traditional health care services and strong support of CHWs as members of the health care team (18,19).
